# Unconfined Compressive Properties of Composite Sand Stabilized with Organic Polymers and Natural Fibers

**DOI:** 10.3390/polym11101576

**Published:** 2019-09-27

**Authors:** Yuxia Bai, Jin Liu, Zezhuo Song, Zhihao Chen, Canhui Jiang, Xiaowei Lan, Xiao Shi, Fan Bu, Debi Prasanna Kanungo

**Affiliations:** 1 School of Earth Sciences and Engineering, Hohai University, Nanjing 210098, China; baiyuxia@hhu.edu.cn (Y.B.); szzhhu@163.com (Z.S.); hhuczh@163.com (Z.C.); Jcanhhhu@163.com (C.J.); lxw_hhu@163.com (X.L.); hhushix@163.com (X.S.); bf_hhu@163.com (F.B.); 2 CSIR-Central Building Research Institute (CBRI), Roorkee 247667, India; debi.kanungo@gmail.com

**Keywords:** unconfined compressive properties, organic polymer, coir fiber, sisal fiber, sand, strength properties, ductile behaviors, microstructure

## Abstract

As renewable and environment-friendly materials, coir and sisal natural fibers can be used in soil reinforcement with minimum cost and other benefits. In this study, we focused on their improvements of unconfined compressive properties of polymer treated sand. In total, 36 groups of unconfined compressive strength tests, combined with X-ray diffraction and scanning electron microscope investigations were performed. We had studied the effects of polymer and fiber contents, and fiber types on the reinforcement effectiveness. The results showed that both coir and sisal fiber can improve the mechanical properties and microstructure of treated sand. In terms of strength properties, sisal fiber inclusion was better than coir fiber, while both have a similar reinforcement benefit on soil ductile behaviors. The strength and compressive energy increased with an increment in polymer and fiber content. The reinforced sand can have up to 1 MPa compressive strength and 140 kPa compressive energy for coir fiber inclusion, while 1.2 MPa and 170 kPa, respectively, for sisal fiber. The axial stress-strain characteristics and failure patterns were also improved, and the brittle index decreased toward zero, which suggests an increasing ductile. The polymer membrane enwrapping and bonding sand grains, and the network structure built by fiber crossing and overlapping among sand grains, as well as the interfacial attachment conferred by polymer between sand grains and fiber, all contributed to the reinforcement of treated sand.

## 1. Introduction

Sand is extensively distributed in Northern China, and some natural disasters, such as foundation failures, road deformations, and soil erosion, are induced by the properties of sand with less clay content and loose structure. Hence, it is necessary to reinforce sand when used in construction and engineering. Currently, cement, slag, and lime are classical chemical additives used to improve the microstructure, strength, and other properties of problematic soil [1,2]. Yoon and Abu-Farsakh [1] perform compaction, unconfined compression, and tube suction tests to investigate the strength properties and water susceptibility of cement-sand, and conclude the cement-sand can be classified as a good quality base material. Short fibers are another common reinforcement material used to enhance the shear strength properties and anti-liquefaction resistance of sand [3,4]. Liu et al. [4] consider the fiber content and sand density impact on liquefaction resistance of sand with undrained ring-shear tests, and find fiber reinforcement is useful for improving the static liquefaction resistance of sand. The laboratory and in-situ test results prove the potential use of fibers for reducing the brittle behaviors of cement-treated sand [5].

However, such classical chemical additives show some drawbacks in environmental protection where high pH inhibits the normal growth of plants [6]. Yet, it is also prone for forming a brittle fracture and reducing the mechanical performance [7]. Therefore, a series of new type additives, such as liquid polymer, resins, acids, silicates, and ions, have drawn increasing attention [8,9]. Polyurethane is a versatile polymeric material, and could be tailored to meet the diversified demands of engineering practices [10]. Zang et al. [11] studied the relationship between the molecular structure of waterborne polyurethane dispersions and the sand fixing effect, considering the effects of diisocyanate-to-polyol ratio and molecular weight. Liu et al. [12] used water-based polyurethane to reinforce the topsoil of sandy soil slop, and the results showed that the polyurethane treatment could significantly increase the strength properties, reduce the permeability, and improve the water ability of the sand. Moreover, polyurethane treatment improved the water retention properties and provided a suitable environment for vegetation growth. These research achievements all demonstrate the extensive prospects of polyurethane applied in soil reinforcement.

Additionally, in view of the advantages of natural fibers, such as accessibility, renewability, low density, lower prices, and satisfactory mechanical properties [13,14], they turn to a more attractive selection than synthetic fiber for the reinforcement of soil mass. Coconut coir fiber is extracted from the husk of coconut (Cocos nucifera), which seeds in tropical parts of the world such as in the Philippines, Indonesia, and India [15]. The global production of this fiber is about 5 million tons per year [16], and this fiber can be either used directly in products, such as in erosion control blankets, or spun into yarn that can be weaved into products [15]. However, coir fiber is also considered a waste material in many countries, such as in India, and it is reported that, for every ton of fiber extracted, about 2 tons of coir waste is produced [17]. Sisal fiber is obtained from the leaves of the sisal plant (Agava sisalana). It is one of the most commonly used natural fibers, mostly attributed to the easy cultivation and short renewal times of sisal plants [18]. These plants are now widely grown in tropical and sub-tropical North and South America, tropical countries of Africa, the West Indies, and the Far East [19]. It is reported that about 4.5 million tons of sisal fiber are produced every year throughout the world [20], and the sisal fiber is widely used in transportation, fishery, petroleum, metallurgy, and other industries. However, a large quantity of this renewable resource is still underutilized. It is also reported that, when the total world produced about 0.6 million tons of sisal fibers, it would accordingly produce about 20,000 tons unutilized and wasted leaves, valued about Rs 5 crores [18]. Engineered utilization of sisal fiber and coir fiber can reduce the disposal problem and environment pollution. Furthermore, the use of natural fibers as construction material offer significant cost reduction and benefits associated with processing. Studies have shown the potential use of sisal fiber and coir fiber to improve the properties of construction materials [21,22].

Nevertheless, the cooperation treatment with chemical stabilization and fiber reinforcement show clear merit on the reinforcement benefit than using the chemical additives or fibers separately [23,24]. Malidarreh et al. [24] investigate the effects of three different fibers (sack, PET, and PP fibers) on the mechanical behavior of artificially cement-treated Babolsar sand, and find that the addition of fibers has a significant effect on the behavior of cemented sand. However, PP fibers are much more effective as reinforcing materials than sack and PET fibers. In this study, the polyurethane, coir fiber, and sisal fiber were used as composite material to reinforce sand, and the addition of coir fiber and sisal fiber on the mechanical properties and microstructure of the sand stabilized with organic polymer were investigated. Unconfined compressive strength measurements were performed on the treated sand to evaluate the mechanical properties. The emphasis was on the effects of polymers content, fibers content, and fiber types. X-ray diffraction tests and scanning electron microscope analysis were also conducted to investigate the micro-interactions of polymer-fiber-sand mixtures. This research provided useful references for the applications of natural fibers in engineering construction, and it suggested an alternative method for soil reinforcement.

## 2. Materials and Methods

### 2.1. Sand

**Figure 1:**
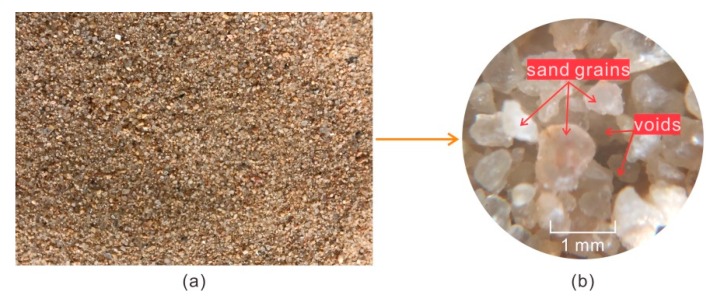
The used sand in this study: (**a**) macro and (**b**) micro.

The sand used in this study is derived from the Nanjing area in Eastern China. The sand taken back from the site was air-dried first, and then sifted with 2 mm sieve. The prepared sand was pictured in Figure 1, and the physical parameters were measured according to the laboratory soil tests displayed in Table 1. As shown, the irregular block sand grains were dis-attached, and the sand showed less clay content and a loose structure. The main component of the sand was the grains in the range of 0.1 to 0.5 mm. The uniformity (*C*_u_) and curvature coefficient (*C*_c_) were calculated as 2.27 and 1.13, respectively. Thus, the sand was defined as poorly graded (SP), according to the Unified Soil Classification System (USCS) (American Society of Testing Materials D2487-11, West Conshohocken, PA, USA).

### 2.2. Organic Polymer

**Figure 2:**
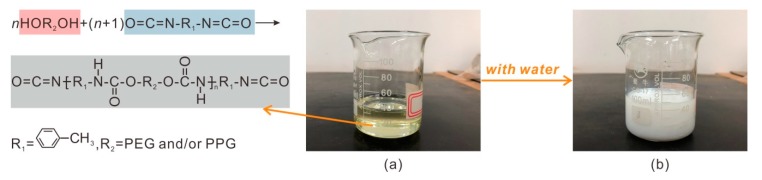
The photographs of (**a**) organic polymer and (**b**) hydrogel.

The organic polymer (OP) selected to stabilize sand contains plenty of polyurethane pre-polymer. The polymer used in this study is prepared according to a fixed procedure, and the reaction formula is given in Figure 2.

First, the 50 g poly-oxypropylene diol (PPG) with molecule weight 2000, 50 g poly-oxyethylene glycol (PEG) with molecule weight 2000, 20 g poly-caprolaclone glycol (PCL) with molecule weight 1000, and 200 mL toluene were added to the flask, and then heated at 145 °C with stirring.

When the polymer polyols were completely melted, the air distillation was performed. Afterward most of the toluene were dried. Vacuum distillation was used to totally remove the residual water and toluene.

Then, the mixture was chilled to 20 °C. After that, the deco-compression device was removed and the reflux condenser was placed. This was followed by the addition of N_2_. The reaction system was sealed with oil. Next, 20 g toluene diisocyanate was added, the sample was heated at 90 °C, and reacted for 2 h.

After that, the mixture was cooled to 20 °C, and added 200 mL ethyl acetate with stirring for an hour. Then, the polyurethane pre-polymer was obtained. Lastly, the lauryl sodium sulphate was mixed with polyurethane pre-polymer in a moderation ratio to achieve the final organic polymer.

As shown in Figure 2a, the organic polymer was slight yellow liquid, with a pH of 7, *G*_s_ of 1.15 g/cm^3^, viscosity of 650 to 800 MPa·s, and solid content of more than 88%. This organic polymer was very reactive to water and formed hydrogels with good viscosity (Figure 2b). The coagulation time of the hydrogel was about 60–1600 s and can be reduced by raising the ratio of the polymer to the water.

### 2.3. Fibers

**Figure 3:**
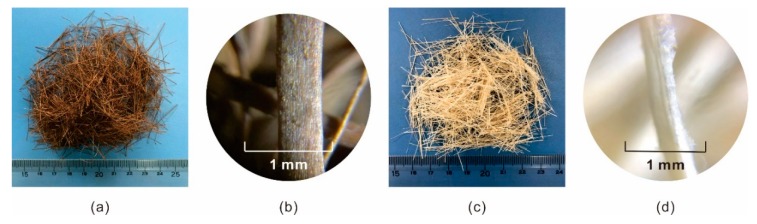
The images of fibers: (**a**) CF macro, (**b**) CF micro, (**c**) SF macro, and (**d**) SF micro.

Coir fiber (CF) and sisal fiber (SF) contain high content of cellulose and lignin. The former is the primary constituent of these fibers, while the latter acts as the cementing agent inside the fiber, which binds the cellulose fibers together [20,22,25,26]. Coir fiber is composed of 37% cellulose and 42% lignin and other compositions, while sisal fiber mainly contains 70% cellulose and 12% lignin [18]. The physical properties of coir fiber and sisal fiber used in this study were provided by the manufacturer (Table 2). All fibers were prepared to be 18 mm in length, as given in Figure 3a,c. Within our test range, these short fibers can be added to the soil randomly and evenly like cement to maintain isotropic strength, and avoid forming potential weak planes in fiber-soil mixtures induced by reinforcement direction and spacing. The fibers were used in the condition that they were received in from the factory without any further treatment. Due to the fibers being obtained from the corresponding plants and with washed treatment [20], there is still surplus waste. Inorganic compounds, sugars, and starches remain on the surface of the fibers [22], as shown in Figure 3b,d. Figure 3b also illustrated there were rich micro-cracks and cell lumens on the surface of the coir fibers.

### 2.4. Composite Specimen Fabrication

In this study, the addition amount of polymer and fiber was calculated according to the weight of dry sand, as shown in Equations (1) and (2) [4,27]. Upon a pre-experimental basis, the content of the polymer was set as 1%, 2%, 3%, and 4%, respectively, while that of the fiber varied from 0% to 0.8% with an increment of 0.2%. The specimens for unconfined compressive strength (UCS) tests were prepared as a cylindrical shape with 80 mm in height and 39.1 mm in diameter using the static compaction method. The specimens were prepared with an initial water content of 10% and a dry density of 1.5 g/cm^3^. The preparation process for organic polymer stabilized sand (OPSS), organic polymer-coir fiber, and organic polymer-sisal fiber reinforced sand (OPCFRS and OPSFRS) was different.

(1) $$C_{op}=\frac{w_{op}}{W_{s}}$$

(2) $$C_{fi}=\frac{W_{fi}}{W_{s}}$$

where $C_{op}$ (%) is defined as the polymer content, $W_{op}$ (g) is the weight of the used polymer, and $W_{s}$ (g) is the weight of dry sand. $C_{fi}$ (%) is defined as the fiber content and $W_{fi}$ (g) is the weight of the used fiber.

**Figure 4:**
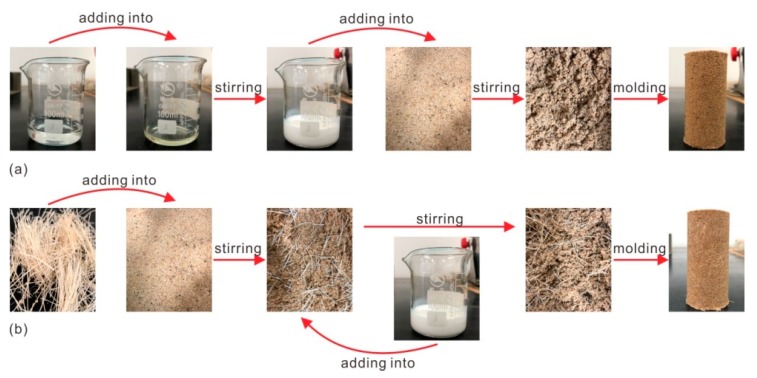
The diagram of the composite specimen fabrication: (**a**) OPSS and (**b**) OPSS with fiber.

For the OPSS, the fabrication process was displayed in Figure 4a. The necessary sand, polymer, and water were prepared with the required quantity first, and then the obtained water was added into the polymer and stirred with a glass rod for about 20–40 seconds to achieve a milky solution. After that, the prepared polymer solution was added into the sand and mixed with a coulter knife for 1–2 min to obtain a uniform polymer-sand mixture. Next, the mixture was transferred into the steel mold and compacted to the pre-determined height by applying static pressure. By comparison, the difference in OPCFRS or OPSFRS preparation was that fibers were added prior to the addition of the polymer solution and mixed manually with wet sand in a small increment to ensure uniform distribution. The fabrication process for the polymer and fiber-treated sand was displayed in Figure 4b.

The specimen was dismantled after being compacted for 2 to 5 min, and placed in a curing box for 2-days curing with a temperature of 27 ± 3 °C. Then, UCS tests were performed.

### 2.5. Testing Method

**Figure 5:**
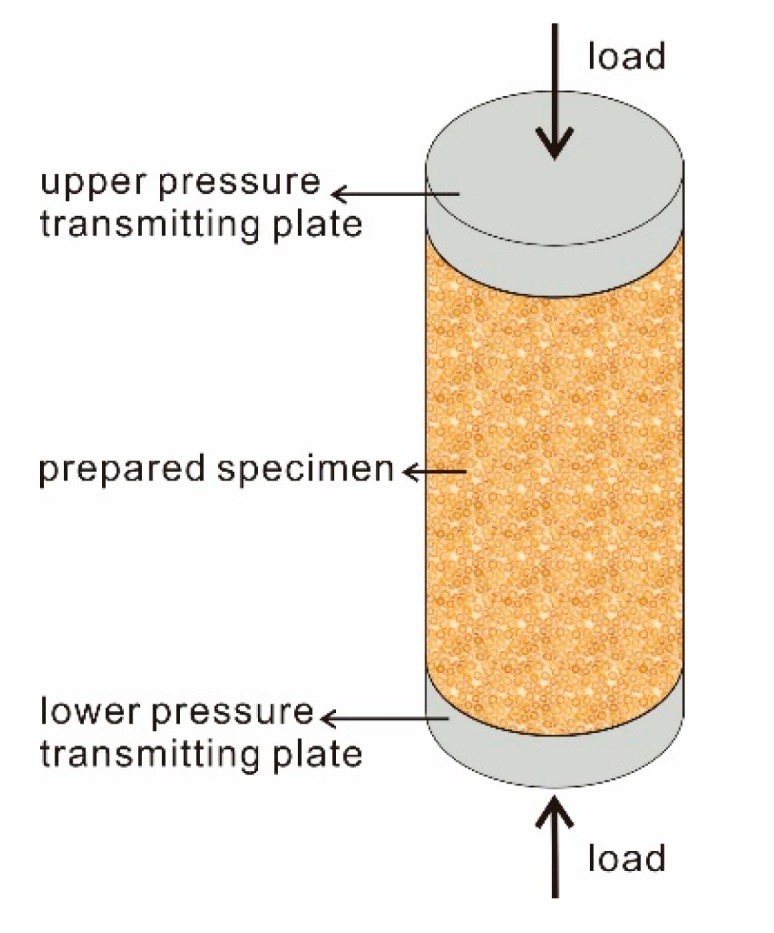
The diagrammatic sketch of the UCS test.

Unconfined compressive strength test is a vital test content in rock and soil mass [27,28]. It is a theoretical test value that the lateral edge of the specimen is not restricted in the experiment. In practice, there is actually a lateral limit for the objects, and the tri-axial test is more realistic compression and shear measurement considering the lateral in-situ stress. Hence, the UCS test is considered a special case of the tri-axial test, and used extensively because of its better convenience of operation and lower economic cost. The test results are of great significance to the design and construction of buildings and engineering. The diagrammatic sketch of the UCS test was shown in Figure 5. As illustrated, the prepared specimen was placed between the upper and lower load transmitting plates. The lateral edge of specimen was in free, that is, the specimen had no confining pressure. The upper and lower load transmitting plates were used to apply the pressure evenly to the specimen. Prior to experimentation, check and ensure good contact existed between the specimen as well as the upper and lower load transmitting plates. Then, the compressive load was applied to the specimen until it failed, and the axial stress-strain curve of the specimen during this process was recorded for further study.

Energy absorption, a useful evaluated parameter for soil reinforcement, indicates the amount of energy required to induce certain deformation of treated sand [5], and the energy absorption is calculated from the area under the stress-strain cover up to the selected strain value [5,29]. In this study, energy absorption was determined to the axial strain of 10% (*E*_10_) and 20% (*E*_20_), according to following Equation (3). It should be noted that the calculated values of *E*_10_ and *E*_20_ are provisional because of the measurement error, and the error is controlled within required range.

(3) $$E=\sum_{i=0}^{i=n}\frac{\left(\sigma_{i}+\sigma_{i+0.25}\right)}{2}\cdot 0.0025$$

where the E (kPa) is the required energy to induce 10% or 20% deformation of treated sand; i (%) is the axial strain, with an increment of 0.25%; n (%) is the selected axial strain (i.e., 10% or 20%); $\sigma_{i}$ (kPa) presents the axial stress corresponding to the i strain; and 0.25 (%) presents the axial strain interval.

In this study, the UCS tests were performed using a standard strain-controlled unconfined compressive apparatus with a load rate of 2.4 mm/min. The axial stress and displacement of the treated sand were recorded, according to the axial dynamometer and axial displacement gauge, respectively, to obtain the axial stress-strain curves, and the measurement process ended until the axial strain reached about 25%. Three measurements in the same case were averaged and used.

## 3. Results and Discussion

A series of UCS tests were performed on the treated sand using polymer, coir fiber, and sisal fiber to investigate the addition of natural fibers on the reinforcement benefits. Several factors that may influence the strengthening effects were considered, such as polymer content, fiber content, and fiber types. Therefore, a total of 36 groups with treated specimens were tested, and the results were summarized in Table 3.

### 3.1. Axial Stress-Strain

**Figure 6:**
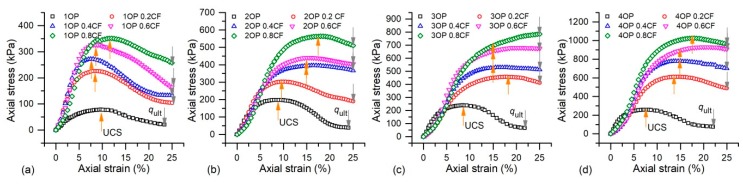
The axial stress-strain curves of OPSS and OPCFRS: (**a**) *C*_op_ = 1%, (**b**) *C*_op_ = 2%, (**c**) *C*_op_ = 3%, and (**d**) *C*_op_ = 4%.

**Figure 7:**
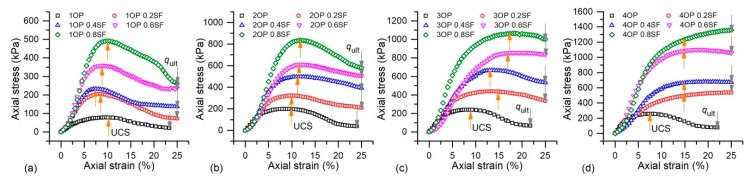
The axial stress-strain curves of OPSS and OPSFRS: (**a**) *C*_op_ = 1%, (**b**) *C*_op_ = 2%, (**c**) *C*_op_ = 3%, and (**d**) *C*_op_ = 4%.

The typical axial stress-strain curves of OPSS, OPCFRS, and OPSFRS were plotted in Figure 6 and Figure 7. It was observed that the initial stress of all specimens was similar, which implies that the polymer and fibers inside the sand matrix had not worked yet. With a further increase in axial displacement, the stresses increased accordingly until they reached a peak, and the ones with fiber exhibited better compressive properties when compared to the others stabilized with a pure polymer. By comparison, the increasing rate and peak value of OPSFRS were both higher than that of OPCFRS, and it may be induced by the excellent elastic modulus of the sisal fiber. Figure 6 and Figure 7 also showed that, after the peak, the post-peak stress did not show a sharp decrease like that of cemented soil, which demonstrated good ductility for polymer and fiber-treated sand. The peak stress of OPSS was obtained at about 7–10% strain, where, that of the cemented soil, was observed at about 2% to 4% [5], due to the good elasticity and flexibility of the thin and hardened membrane formed by the polymer among sand grains. Moreover, the coir fiber addition resulted in an increase in the strain corresponding to peak stress, up to about 15%. The fiber reinforcement benefit was influenced by the polymer content (Figure 6a–d), and higher polymer and fiber content induced the stress-strain to a work-hardened form (Figure 6c,d). It was expected that the fiber presence resisted grains displacement and impeded crack propagation via interface friction and interlinking among sand grains. Due to the bond strength conferred by the membrane between sand grains and fiber, it would further enhance the sand grain-fiber interfacial force and, therefore, led to an improvement in the fiber reinforcement effect. Moreover, the higher polymer content led to stronger bond strength. The similar phenomenon was also observed in treated sand with sisal fiber (Figure 7a–d). However, there was no clear difference on stress-strain characteristic improvement between OPCFRS and OPSFRS, which may be because of the similarity on softness and interlinking ability of the coir and sisal fiber.

### 3.2. Unconfined Compressive Strength

As introduced above, there are two different axial stress-strain curves forms of the treated sand. For the first one with the clear peak value, the peak stress is defined as UCS, and for the latter without a peak value, the axial stress corresponding to the strain of 15% is used as UCS for further study, as shown in Figure 6 and Figure 7.

**Figure 8:**
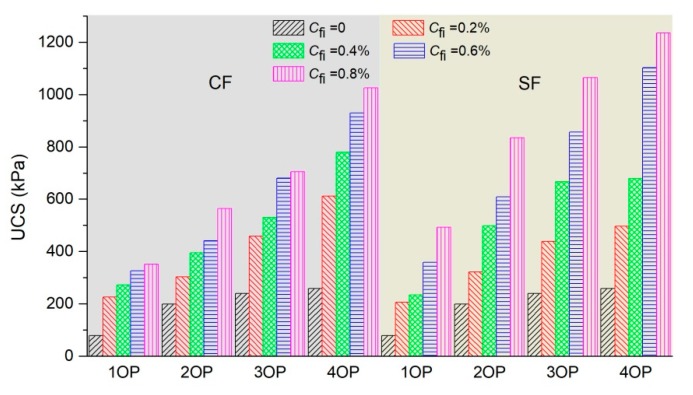
The UCS of OPSS, OPCFRS, and OPSFRS.

The UCS of OPSS, OPCFRS, and OPSFRS were summarized in Table 3 and Figure 8. As displayed, the UCS of treated sand increased with an increment in polymer content. It was expected that an increase in polymer content would enhance the membrane effect by increasing the coating soil area and bonding strength. Moreover, the UCS of treated sand always increased when the polymer content increased regardless of how much fiber was added, and the UCS increment induced by the polymer content grew with the addition of fiber and fiber content. The variation of the polymer content from 1% to 4% led to an increment in UCS with 179 kPa for pure polymer reinforcement and 673.82 kPa for polymer and coir fiber cooperation treatment (Table 3) respectively, which also proved to have a great treatment benefit induced by cooperation reinforcement. The polymer and fiber treatment led to more improvement in UCS, which can be explained by an addition to the polymer membrane enwrapping and bonding soil grain, and the interfacial force between the sand grain and fiber. The bond built by the membrane between sand grains and fibers also contributed to the improvement of the cooperation treatment effectiveness. Figure 8 also showed that the fiber content increase led to an enhancement in compressive strength, and the treated sand with coir fiber yielded up to about 1 MPa compressive strength, with 1.2 MPa for sisal fiber cooperation treatment (Table 3). Moreover, the sisal fiber illustrated a better effect for the improvement of compressive strength than the coir fiber. For example, the 0.8% coir fiber addition resulted in the UCS of 4% polymer reinforced sand increasing by four times, while the treated sand with sisal fiber increased by 4.8 times. Except for the excellent elastic modulus of sisal fiber, the residual substance on the surface of sisal fiber may also strengthen the interaction force between the sand grain and fiber, and it was, therefore, the sisal fiber cooperation treatment that demonstrated better reinforcement.

### 3.3. Energy Absorption

In order to take further investigation on the effect of fiber addition on the unconfined compressive characteristics, the energy absorption is also used to evaluate the reinforcement effects of composite sand. 

**Figure 9:**
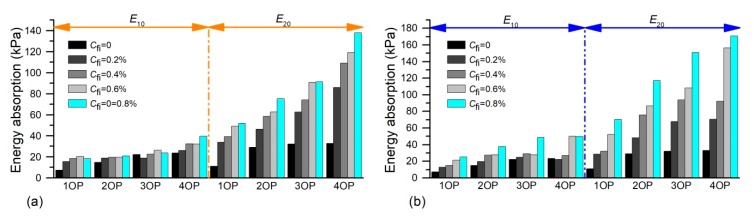
The variation of *E*_10_ and *E*_20_ of treated sand with polymer and fiber contents: (**a**) OPSS and OPCFRS. (**b**) OPSS and OPSFRS.

The calculated *E*_10_ and *E*_20_ according to Equation (3) were given in Table 3 (shown in the beginning of the Results and Discussion part), and the relationship between absorbed energy and polymer and fiber content was given in Figure 9. It was observed that *E*_10_ and *E*_20_ both increase when the polymer content increases. The *E*_10_ of treated sand with different polymer content (i.e., 1–4%) was observed as 7.33, 15.06, 22.39, and 23.63 kPa, respectively (Table 3). The difference of *E*_10_ induced by the polymer amount implied that the presence of the polymer has worked before the axial strain reached up to 10%, and the higher polymer content treatment needed more energy to induce an equivalent deformation. Figure 9 also showed that the *E*_10_ and *E*_20_ value both increased with the fiber content increasing. Nevertheless, the fiber content played a more vital role on *E*_20_ than that on *E*_10_. For example, 0.8% sisal fiber addition led to *E*_10_ and *E*_20_ of 4% polymer-treated sand increasing by 26 and 173.65 kPa, respectively (Table 3). It was expected that the fiber addition improved the bearing capability of treated sand and, therefore, the fiber effect was not fully apparent before the axial strain increased up to 10%. However, the specimen may have failed before 20% deformation, and the fiber effect had been fully exerted, which results in apparent difference in absorbed energy. Moreover, the sisal fiber had greater advantages than coir fiber on improving energy absorption. It needed a 39.71 and 137.90 kPa induction of 10% and 20% deformation for OPCFRS, while it was 49.63 and 170.52 kPa for OPSFRS (Table 3). This may be attributed to the excellent elastic modulus of sisal fiber.

**Figure 10:**
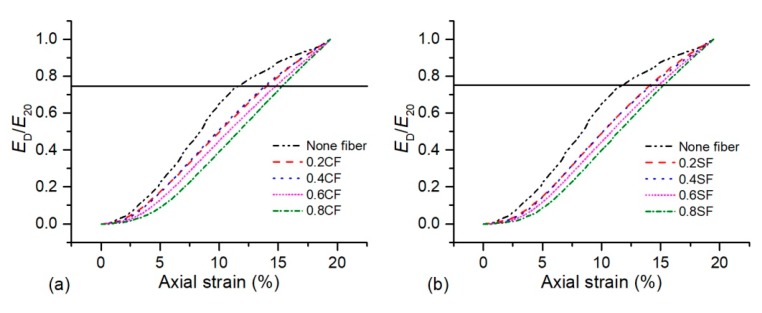
The normalized absorption energy curve of the polymer-treated sand with (**a**) CF and (**b**) SF.

Absorbed energy for different axial strains (*E*_D_) is normalized to the energy absorbed in the 20% axial strain for different fibers content [5,30], which is used to further investigate energy absorption. The normalized absorption energy curve of 1% OP-treated sand with coir and sisal fiber was shown in Figure 10. As observed, the slope of the energy absorption ratio curve for OPSS changed, and an apparent change in the slope can be observed at about 75% energy absorption and 11% axial strain. The literature found that a breakdown in the rate of the normalized energy absorption curve for fiber-reinforced cemented sand was observed at an energy absorption of 50% [5] and 30% [30]. This difference may be induced by the elasticity and flexibility behaviors of reinforcement materials. However, the inclusion of fibers and an increment in fiber content both led the slope of the curves to flatten, and it meant that the energy absorption capacity of the treated sand increased at a constant rate. When the fibers content increased from 0% to 0.8%, the associated axial strain increased to about 15% regardless of the fiber types. Furthermore, the slope of the energy absorption ratio curve was proactively unchanged by the fiber types.

### 3.4. Brittle Behaviors

The most impressive advantages of the fiber reinforcement when applied to cemented soil are the improvement in ductility. The brittle index (*I*_B_) determined by peak stress and residual or ultimate stress of stress-strain, showing the forms of treated soil, is used widely to study the ductile behaviors of treated soil [30,31]. In this study, *I*_B_ was calculated by the following equation.

(4) $$I_{B}=\frac{UCS}{q_{ult}}-1$$

where $I_{B}$ (dimensionless) is the brittle index, and, as the $I_{B}$ value decreases toward zero, the failure behavior becomes increasingly ductile. $q_{ult}$ (kPa) indicates the ultimate stress of the axial stress-strain curve, as shown in Figure 6 and Figure 7.

**Figure 11:**
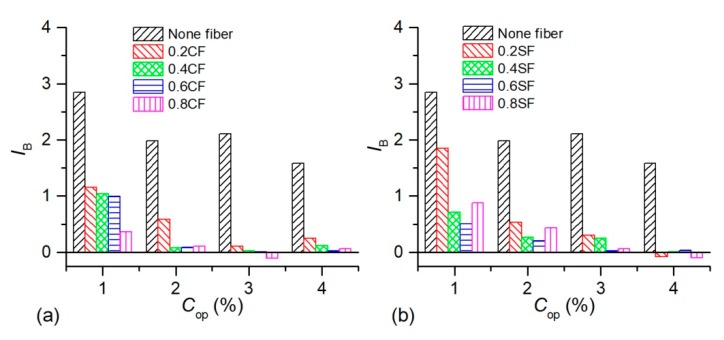
The variation of *I*_B_ of treated sand with the addition amount of polymer and fibers: (**a**) OPSS and PCFRS. (**b**) OPSS and OPSFRS.

The calculated *I*_B_ values of OPSS, OPCFRS, and OPSFRS were given in Figure 11. As shown, the *I*_B_ value of treated sand decreased with the polymer content increasing. It also displayed that an increase in polymer content always led to the *I*_B_ value reducing no matter how many fibers were added. The improvement in soil ductile behavior was due to the good elasticity and flexibility properties of the polymer membrane, and it played as a buffer among sand grains to avoid the suddenly failure of cemented soil. Moreover, the buffer effect was influenced by polymer content, and grew with an increase in polymer amount. Figure 11 also illustrated that the fiber inclusion and fiber content both increasing led to a reduction in the *I*_B_ value. For example, the 0.8% coir fiber inclusion resulted in an *I*_B_ value of 3% OP-treated sand decreasing from 2.11 to −0.10, and that of sisal fiber cooperation treatment decreasing from 2.11 to 0.06. These were due to the three-dimensional fiber structure resisting sand grains displacement and impeding crack propagation. This would provide residual stress via the bridge effect [28,32]. Furthermore, the higher the addition amount of the polymer, the more remarkable the fiber reinforcement effect. Several treated specimens’ *I*_B_ value were lower than zero, showing a work-hardened form. This means an increase of ductility. These improvements indicated that the cooperation of the polymer and fiber on the enhancement of soil behavior was better than using one of them separately. Additionally, with respect to the improvement on soil ductile behavior, the coir fiber and sisal fiber had similar reinforcement effects. None of them showed clear advantages.

### 3.5. Failure Pattern

**Figure 12:**
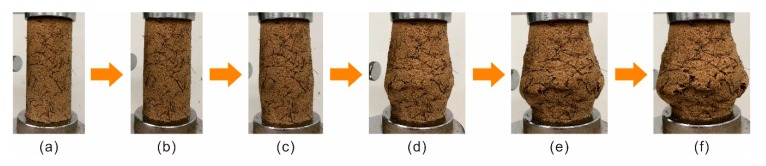
The photographs of treated sand during the UCS test process in (**a**) *ε* = 0%, (**b**) *ε* = 5%, (**c**) *ε* = 10%, (**d**) *ε* = 17%, (**e**) *ε* = 23%, and (**f**) ultimate.

The photographs of treated sand during the UCS test were recorded in Figure 12. During the initial stage, the prepared specimen was being compressed. Hence, there were no apparent changes in specimen shape, besides a slight variation in height, as shown in Figure 12a,b. With an increment in compressive load, due to the reduction of vertical space and the lateral space of the specimen being in a free state, bulging occurred along the middle section of the specimen (Figure 12c). Furthermore, the bulging tendency increased with axial deformation increasing. The bulging form may be induced by the elasticity and ductility of the polymer membrane inside the specimen. When the applied load reached the critical value, the polymer membrane and fiber reinforcement began to lose effectiveness gradually. Some micro-cracks appeared along the surface of the specimen (Figure 12d), and then evolved into macro-cracks with an apparent length and width (Figure 12e) with axial deformation further increasing. Lastly, this results in thorough failure (Figure 12f). Herein, the final state of the treated sand after performing the compressive tests is defined as a failure pattern.

**Figure 13:**
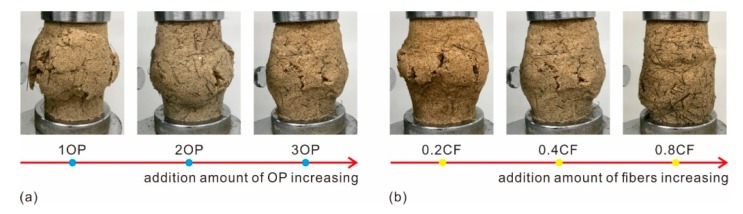
The failure pattern of OPCFRS with (**a**) 0.4CF and different OP contents. (**b**) 3OP and different CF contents.

**Figure 14:**
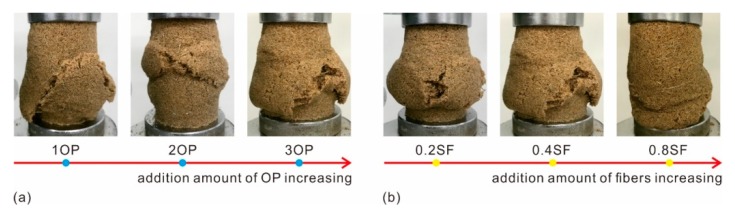
The failure pattern of OPSFRS with (**a**) 0.4SF and different OP contents. (**b**) 3OP and different SF contents.

The failure patterns of OPCFRS and OPSFRS using different polymer and fiber contents were shown in Figure 13 and Figure 14. As expected, the specimens all destroyed the middle section. As shown in Figure 13a and Figure 14a, the treated specimens with 1% OP were divided into several segments by macro cracks and these cracks may connect somehow inside the specimen. Moreover, the failure forms got better with polymer content increasing. The macro cracks that formed along the surface of the specimen became narrow and short (Figure 13a and Figure 14a), and reduced the connection possibility among these cracks, and, therefore, improved the completeness of treated sand. Figure 13b and Figure 14b also showed that an increment in fibers content resulted in significant improvement in soil ductile behaviors visually. For the treated sand with little fiber addition, there were still macro cracks on the surface of the specimen. Nevertheless, with fiber content increasing, the visible cracks on the surface of the specimen became micro-cracks. The failure form of treated sand with higher fiber content bulged and twisted (Figure 13b with 0.8CF and Figure 14b with 0.8SF). Since the distribution density increasing induced by fiber content was more effectively disturbing the sand grains displacement to the free side or space [32], the sand grains consumed the external energy by moving or flipping within the limited space inside of the specimen, with ineffective displacement [27].

Because of the previously mentioned descriptions, there are two different failure models of treated sand including the sliding-petal model as well as the bulging-twisting model. The first model (sliding-petal) commonly appeared in treated sand with little polymer and fiber content and the specimen was divided into several segments by macro cracks. The second (bulging-twisting) refers to the specimen characterized by bulging and twisting, without clear cracks. In addition, the specimen reinforced with higher polymer and fiber content destroys these characteristics.

## 4. Cooperation Reinforcement Mechanism

**Figure 15:**
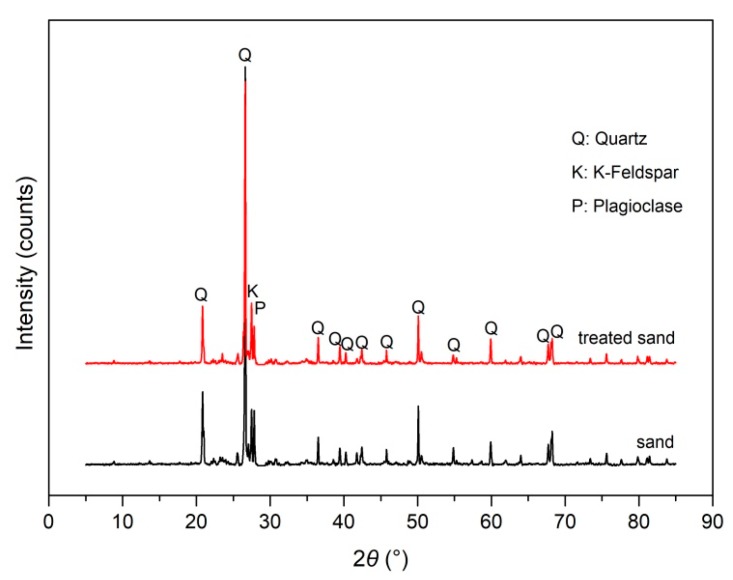
The XRD tests results of (**a**) sand and (**b**) polymer-treated sand.

**Figure 16:**
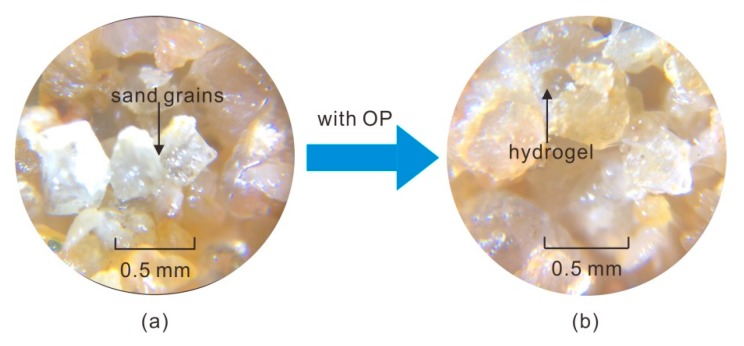
The micro photographs of (**a**) sand and (**b**) OPSS.

The XRD results of sand and treated sand were displayed in Figure 15. As observed, there were no new materials in treated sand, which generated because of the addition of the polymer. Generally, there was no chemical reaction between polyurethane (i.e., the products of water and polymer) and sand grains. The hydrogel, formed by the polymer and water, absorbed on the surface of sand grains via hydrogen and inter-molecular force, and the hydrogel gradually turned to a thin and hardened polymer membrane with the evaporation of water, which resulted in an improvement in elasticity, flexibility, and strength [27,28,33]. The micro photographs of sand and polymer-treated sand were given in Figure 16. As shown, the unreinforced sand with a loose structure and small adhesive force was not prepared as the standard cylindrical specimen. The polymer addition effectively improved this issue. The polymer hydrogel enwrapped the sand grains and connected the dis-attachment sand grains, which led to an increment in adhesion among soil grains and a reduction in soil voids, like that shown in Figure 16b, and it, therefore, enhanced the stability of the micro-structure inside the specimen.

**Figure 17:**
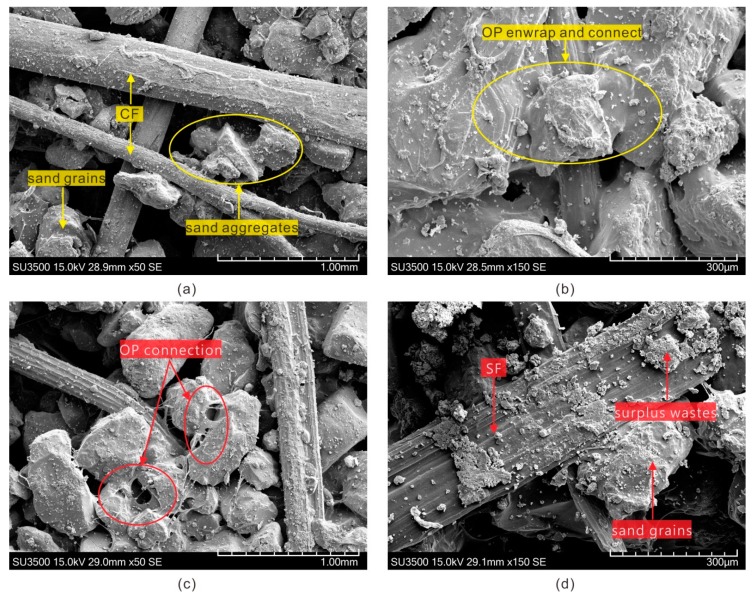
The SEM photographs of (**a**) OPCFRS with 50 magnification, (**b**) OPCFRS with 150 magnification, (**c**) OPSFRS with 50 magnification, and (**d**) OPCFRS with 150 magnification.

The micro structure of treated sand was studied by scanning electron microscopy (SEM, SU3500, Hitachi, Tokyo, Japan), and the SEM photographs of OPCFRS and OPSFRS were shown in Figure 17. Due to the presence of the polymer, the dis-attachment sand grains easily formed sand aggregates (Figure 17a). It also showed that the distributed natural fibers crossed and interlinked to build a three-dimensional structure among sand aggregates to restrain the displacement of sand grains (Figure 17a,c). With the high hydrophilic properties of natural fibers, the polymer hydrogel was easily attached on the fibers’ surface to provide a good bond between the fiber and sand grain (Figure 17b,c), which resulted in an improvement of the micro structure in a sand-fibers matrix. However, the residual material on the fiber surface would influence the interactions between the fiber and the polymer membrane, which leads to a reduction in bond strength. However, at the same time, the residue would increase the friction strength between the fiber and sand grain somehow. Additionally, the surplus residue on the surface of sisal fiber was more clear than that on the coir fiber (Figure 17b,d). Moreover, when the polymer solution mixed with the sand-fiber matrix, the polymer solution would flow into the cracks and cell lumens on the rough coir fiber surface and then consolidated to form a bond. Therefore, the contributions to the improvement of the micro structure of treated sand can be concluded as [28,33]: (i) the polymer membrane coating and bonding sand grains, (ii) the interfacial physical force between the sand grain and fiber, and (iii) the bonding conferred by the membrane between the fiber and soil grain. Furthermore, the polymer content and fiber content increasing both improved these introduced aspects.

It was expected that the addition of the polymer and fiber would lead to a reduction in voids, which contributed to improving mechanical properties. Figure 17 also showed that, even though the polymer and fiber built a dense network structure among sand grains, the voids were not fully filled with reinforcement materials, and there were still voids inside the treated sand mass. On the basis of the porous properties, the presence of the polymer and fiber improved the cohesion between sand grains by avoiding the soil erosion. Both would provide a suitable environment for vegetation growth.

As analyzed above, the presence of the polymer actually played a significant role on the composite reinforcement benefit with respect to the unconfined compressive properties. First, the treated sand showed an initial stable micro structure with good membrane connections and fiber network structure. When there was a compressive load, the specimen was compressed first, which means the sand grains made an available displacement within the limited space, which results in the partial membrane being in tension and some being in compression. During this process, the previously free fibers were turned to an in-stress state, such as the flattened turned to bending. When the compressed membrane reached the limits, the sand grains would begin sliding gradually and led to the compressed membrane becoming tense. With the compressive load further increasing, the membrane connections were mostly tense and the natural fibers were mostly in a stress state. When the displacement potential of sand grains exceeded the bearing capability of the polymer membrane, the membrane breakage occurred along where the connection was weakest, or the membrane attachment fell off from the surface of the fiber or sand grain. Then, the failure of the specimen began. An increase in polymer content would result an increase in membrane bond strength. Therefore, this would enhance the bearing capability of the membrane and delay the failure. The first membrane failure would cause the surrounding neighbors to fail continuously, resulting in the failure area increasing and micro-cracks appearing. However, the neighborhood natural fibers would resist the displacement and slide of the sand grains to impede the further failure of the specimens. The resistance provided by the fibers was influenced by fibers using the amount, fiber surface roughness, elastic modulus, and other factors. Since the high elastic modulus, the sisal fiber showed better advantages than the coir fiber with respect to improving the strength properties of treated sand. While, in terms of soil ductile behaviors, the coir fiber and sisal fiber displayed a similar reinforcement benefit. When the displacement potential of sand grains exceeded the resistance, the cracks occurred and propagated until the sand grains were in a new balance.

## 5. Conclusions

This study focuses on the addition of coir and sisal fibers for the improvements of unconfined compressive characteristics and the microstructure of polymer stabilized sand. A series of unconfined compressive tests, X-ray diffraction tests, and scanning electron microscope investigations are performed on OPSS, OPCFRS, and OPSFRS. Important conclusions can be drawn as follows.

The inclusion of natural fibers considerably enhances the strength properties. Such an increase is proportional to the increase of polymer and fiber contents. The UCS of reinforced sand could reach up to 1 MPa for OPCFRS and 1.2 MPa for OPSFRS, respectively. The increase of polymer and fiber dosage both resulted in an increase in *E*_10_ and *E*_20_, while *E*_20_ increased more significantly. Moreover, the presence of natural fibers led to the energy absorption capability of treated sand increasing at a constant rate. In terms of strength properties, sisal fiber was comparatively the better alternative than coir fiber.

The presence of natural fibers also effectively improved the soil behaviors of treated sand. An increase in polymer fiber contents both resulted in the axial stress-strain changing to a hardened form, which suggests increasingly ductile behaviors. In addition, the brittle index of treated sand reduced when using the polymer and the fiber amount increased. The lowest was up to zero. The failure pattern of a treated specimen also improved from failure by dividing into several segments by macro-cracks to damage with bulging and twisting. Furthermore, the coir fiber and sisal fiber showed a similar reinforcement effect on soil ductile behaviors.

The polymer reinforcement was attributed to the polymer membrane enwrapping and bonding sand grains to generate a spatial network membrane structure. The fiber reinforcement was induced by the interfacial forces between sand grains and fiber. In addition to the polymer and fiber reinforcement advantages, the improvement in interaction conferred by the membrane between the sand grain and fiber also contributed to the cooperation reinforcement. The reinforcement benefit was influenced by polymer and fiber content as well as fiber characteristics.

## Figures and Tables

**Table 1:** The physical parameters of the sand used in this study.

Properties		Value
**Specific Gravity *G_s_***		2.65
Maximum dry density *ρ_max_* (g/cm^3^)		1.69
Minimum dry density *ρ_min_* (g/cm^3^)		1.33
Weight percentage content (%)	2–1 (mm)	0.2
	1–0.5 (mm)	17.1
	0.5–0.25 (mm)	48.9
	0.25–0.1 (mm)	31.7
	0.1–0.075 (mm)	2.1
Coefficient of uniformity *C_u_*		2.27
Coefficient of curvature *C_c_*		1.13
Soil classification (USCS)		SP

**Table 2:** The physical and mechanical properties of coir and sisal fibers.

Property	Coir Fiber	Sisal Fiber
Length (mm)	18	18
Diameter (mm)	0.1–0.5	0.1–0.3
Density (g/cm^3^)	0.67–10.00	0.75–10.70
Tensile strength (MPa)	108.26–251.90	227.80–1002.30
Modulus of elasticity (GPa)	2.50–4.50	10.94–26.70

**Table 3:** The test results of OPSS, OPCFRS, and OPSFRS.

Number	*C*_op_ (%)	*C*_fi_ (%)	OPCFRS			OPSFRS		
			UCS (kPa)	*E*_10_ (kPa)	*E*_20_ (kPa)	UCS (kPa)	*E*_10_ (kPa)	*E*_20_ (kPa)
G1	1	0	77.93	7.33	11.13	77.93	7.33	11.13
G2	1	0.2	225.57	15.67	33.84	205.38	13.08	28.74
G3	1	0.4	271.53	18.57	39.22	233.64	14.81	32.37
G4	1	0.4	326.14	20.45	49.15	358.64	21.42	52.26
G5	1	0.8	351.14	18.36	51.76	492.29	25.42	70.18
G6	2	0	197.96	15.06	29.12	197.96	15.06	29.12
G7	2	0.2	302.57	19.01	46.24	321.14	19.66	48.38
G8	2	0.4	394.87	19.68	58.46	497.67	27.57	75.87
G9	2	0.4	440.01	19.63	62.50	608.82	27.76	86.65
G10	2	0.8	563.73	21.12	75.24	833.62	37.91	117.01
G11	3	0	238.65	22.39	32.20	238.65	22.39	32.20
G12	3	0.2	457.29	18.91	62.76	438.25	25.07	67.89
G13	3	0.4	530.36	22.59	74.32	666.90	29.23	93.90
G14	3	0.4	679.78	26.55	90.59	856.12	28.09	108.11
G15	3	0.8	703.82	23.80	91.45	1065.06	48.78	150.94
G16	4	0	257.92	23.63	32.87	257.92	23.63	32.87
G17	4	0.2	611.79	26.15	85.86	496.52	22.17	70.78
G18	4	0.4	779.58	32.57	109.02	678.63	26.98	92.08
G19	4	0.4	928.92	32.08	119.02	1101.49	50.11	156.62
G20	4	0.8	1024.96	39.71	137.90	1234.57	49.63	170.52
